# Crisis Resource Management in the Delivery Room: Development of Behavioral Markers for Team Performance in Emergency Simulation

**DOI:** 10.3390/ijerph15030439

**Published:** 2018-03-03

**Authors:** Fabrizio Bracco, Gabriele de Tonetti, Michele Masini, Marcello Passarelli, Francesca Geretto, Danilo Celleno

**Affiliations:** 1 Department of Education Science, University of Genoa, 16128, Italy; masini@vie-srl.com; 2 Valorizzazione Innovazione Empowerment s.r.l., Spinoff of the University of Genoa, 16129, Italy; 3 High Intensive Care Department, Istituto di Ricovero e Cura a Carattere Scientifico Giannina Gaslini Children’s Research Hospital, 16147, Italy; detonetti@gmail.com; 4 National Research Council of Italy, 16149, Italy; passarelli@itd.cnr.it; 5 Department of Educational Sciences, University of Genoa, 16128, Italy; cesca.geretto@gmail.com; 6 Anesthesiology and Intensive Care Department, M.G. Vannini Hospital, Rome, 00177, Italy; danilocell@libero.it

**Keywords:** Crisis Resource Management, obstetric hemorrhage, non-technical skills, High Fidelity Simulation, delivery room

## Abstract

Human factors are the most relevant issues contributing to adverse events in obstetrics. Specific training of Crisis Resource Management (CRM) skills (i.e., problem solving and team management, resource allocation, awareness of environment, and dynamic decision-making) is now widespread and is often based on High Fidelity Simulation. In order to be used as a guideline in simulated scenarios, CRM skills need to be mapped to specific and observable behavioral markers. For this purpose, we developed a set of observable behaviors related to the main elements of CRM in the delivery room. The observational tool was then adopted in a two-days seminar on obstetric hemorrhage where teams working in obstetric wards of six Italian hospitals took part in simulations. The tool was used as a guide for the debriefing and as a peer-to-peer feedback. It was then rated for its usefulness in facilitating the reflection upon one’s own behavior, its ease of use, and its usefulness for the peer-to-peer feedback. The ratings were positive, with a median of 4 on a 5-point scale. The CRM observational tool has therefore been well-received and presents a promising level of inter-rater agreement. We believe the tool could have value in facilitating debriefing and in the peer-to-peer feedback.

## 1. Introduction

The number of adverse events in obstetrics is dramatically relevant because of to the complexity of the operational environment. Up to 5% of obstetric cases are characterized by injuries or even death of the patient, due to factors that could have been prevented or mitigated [1,2]. Among these contributing factors, poor communication and ineffective teamwork contribute to the vast majority of adverse outcomes [3]. Clinical errors are mainly due to team, system, or process failure, rather than individual mistakes [4]; as a consequence, any training oriented to reduce clinical errors should address interprofessional teams [5]. Working as a team requires the proper integration of three kinds of skills: (i) professional skills, i.e., the set of technical knowledge and competencies that are typical of each profession; (ii) cognitive skills, i.e., the capacity to understand the situation and decide accordingly; (iii) interpersonal skills, i.e., the capacity to communicate, coordinate, and cooperate as a team. These three skills are mutually interdependent for safe management of the clinical situation: a lack in one or two of them will result in poor management and a high potential for error and adverse outcomes.

In recent years, a growing body of evidence has demonstrated the importance of the cognitive and interpersonal skills for the clinical practice and how a structured intervention in the training and analysis of clinical processes in terms of cognitive and interpersonal skills can lead to better teamwork and a reduction of adverse patient outcomes [6,7,8,9]. This structured approach has been labeled Crisis Resource Management (CRM) and has been initially developed in aviation as Crew Resource Management. The approach has been recently adapted for Anesthesiology [10,11], and has then been applied to many other medical domains [7]. Key CRM skills embrace problem solving and team management, resource allocation, awareness of environment, and dynamic decision-making [12,13]. These areas encompass a more detailed range of skills that vary in their number, according to specific domain they are applied to and their level of generality.

High Fidelity Simulation (HFS) is one of the most effective methods for training CRM skills [14,15]. It can reproduce critical situations upon which practitioners can have a proper debriefing aimed at fostering metacognition on technical, cognitive, and interpersonal skills that are implicitly performed during everyday activity but that need a clear and conscious focus in order to be trained and promoted [5]. The real challenge in training CRM principles with HFS is to address specific and observable behavior, setting clear criteria for what is considered a good or poor performance [15,16]. For this reason, each skill has to be described in terms of a specific behavioral marker representing what can be observed in a simulated scenario or in real life.

The points listed in the CRM skills are good guidelines for the effective management of a critical situation, however they do not provide enough support for the debriefing after the simulation for two main reasons. First of all, some of the points are very broad and generic (e.g., “Exercise leadership and followership with assertiveness”) and they need a clear and unambiguous definition in order to be used as a criterion for performance observation. Ratings and comments may be very heterogeneous about the same behavior, if the observers do not have a clear and specific definition of assertive leadership and followership. Secondarily, some points are not easily observable because they are related to mental processes (e.g., “Allocate attention wisely”). A proper behavioral marker should explicit an observable action, the explicit result of that very mental process. For these reasons, the CRM points should be accompanied by a specific and observable set of behavioral markers. This is the rationale behind the development of several tools based on behaviorally anchored rating scales (BARS), where the Likert scale is anchored with examples of effective and ineffective behaviors. This kind of scales is easy to use for non-expert observers (as in the peer-to-peer assessment paradigm) and enhances inter-rater reliability [17]. Tools based on BARS have been extensively adopted in medical education both for technical and non-technical skills [18,19], while none has been developed yet taking into account the CRM points.

To the best of our knowledge, in the literature about CRM there is only one study where behavioral markers are applied to obstetric teams involved in emergency simulations [14]. However, this study reports the adoption of a rating form where the CRM key skills were not explicitly overlapping the list provided by Gaba and colleagues and, most of all, it reported only a checklist of actions to be achieved, without the description of a poor performance, as typical of many observational tools concerning non-technical skills. Other studies were based on the CRM principles for teamwork in the delivery room [20,21,22,23], but we did not find evidence for the adoption of a structured observational form of specific behavioral markers. This method was adopted in [24], but the observational form, called MINTS-DR (Multi-professional Inventory for Non-Technical Skills in the Delivery Room) was concerning non-technical skills in the delivery room in general, and not explicitly focused on the CRM. In addition, the number of behavioral markers listed in MINTS-DR was quite high, resulting in a time-consuming tool to use during the debriefing.

In order to fill this gap, and provide a quicker tool for peer-to-peer observation, we decided to develop an observational tool with specific behavioral markers for team performance in a delivery room simulated emergency inspired by the CRM key points. We wanted this tool to be quick to administer, easy to understand for practitioners inexperienced in human factors, and useful for fostering metacognition. In addition, we wanted to use this tool not only as a guide for the debrief after the simulation, but also as a checklist for observers taking part in the training. As demonstrated in a previous study [25], a proper debriefing after the simulation can foster CRM skills not only for those who took part to the scenario, but also for the observers. The observer will therefore become an active agent of the simulation. The learning objectives would change: not only training practitioners to technical and non-technical skills, not only training them to metacognition and reflection upon one’s own actions, but also training them to peer-to-peer observation and feedback in everyday operations. We argue that a non-judgmental peer-to-peer feedback is a good opportunity to learn CRM skills, promote metacognition and reflection upon one’s own practice. An observational tool based on specific and observable behavioral markers could therefore help both those who took part in the simulation, and the colleagues observing the scenario. Moreover, the list of CRM skills should provide both positive and negative examples, in order to help the practitioner to have a range of possible nuances of the behavior. The list should be easy to administer, to understand, and most of all, easy to keep in mind while working or when discussing an event.

## 2. Materials and Methods

The development of the observational tool followed several steps divided into two main stages: tool design and tool testing. We first listed the 15 points of CRM, as provided by Gaba and colleagues [13], together with an extensive description of each of them. For each point, we reported the behavioral markers we already developed in the MINTS-DR [24], a set of non-technical skills for anesthetists, gynecologists, midwives, and assistants working in the delivery room. We distributed across the 15 CRM points the best matching behavioral markers, accounting for skills like leadership, communication, situation awareness, decision making, task management, and teamwork.

After that, we conducted a series of meetings with anesthetists, gynecologists, midwives, and assistants in order to define the specific behavioral marker for each CRM point. We followed the case study method as a knowledge elicitation technique [26]. Each point was first defined according to Gaba and colleagues [13], in order to help practitioners understand its core meaning. As a case study, we then showed the practitioners videos of simulated scenarios of peripartum hemorrhage and asked them to comment the team performance in order to familiarize them with the CRM points. Once the simulations were described in terms of CRM principles, we engaged practitioners in a brainstorming session to provide the best descriptive, observable, and specific behavior for each one of the 15 points, thinking about the activity in the delivery room. We tried to limit the number of items and identify the most descriptive behavioral marker for each point, because we wanted the tool to be rapid and suitable for debriefing after the scenario. We split some CRM points only when the point was double (e.g., Exercise leadership and followership with assertiveness), or was too general to be covered with only one item (e.g., Communicate effectively).

In line with BARS methodology, each behavioral marker was then defined both in positive and in negative terms, i.e., mentioning the behavior representing the best implementation of the CRM skill, and the behavior representing an extremely poor or even absent skill. The two behavioral descriptions were then located at the extremes of a four-point scale. The reason for this choice is to be found in the need for observers to have a clear anchor to understand and assess the observed behavior, with the two extreme points representing the best and worst condition, and the two inner points representing an acceptable and a scarce behavior. We decided to avoid items referring to actions that may have not been observed and therefore not being applicable to the current scenario (e.g., “if the treatment is not effective, the team can change the therapeutic plan”). Additionally, in our experience, the conditional expression (“if… then…”) is not easy to understand and to observe: even when the condition takes place, the observers may disagree on the truth-value of the precondition itself.

In addition, we decided to interpret each CRM point by taking into account the team as a whole. Therefore, the behavioral markers we provided could be applicable to any professional working in the delivery room. Since some of the CRM points are quite generic (e.g., “Communicate effectively”), some of them had more than one behavioral marker. The final list of items is presented in Table 1.

After the development of the behavioral markers list, we also produced a sheet with short descriptions of the 15 CRM points. We ended up with a booklet (see the supplementary material) that was given to each participant in the second stage of the research: testing the tool. The experimental design for the data collection was approved by the ethical committee (ethical code number: 005) of the Department of Education Sciences of the University of Genoa.

Testing the tool involved six teams working in the obstetric ward of six different Italian hospitals (N = 52). Each team was composed by anesthetists (N = 14), gynecologists (N = 12), neonatologist (N = 1), midwives (N = 14), nurses (N = 5), and the risk manager (N = 6). All of them were informed about the research, they signed a consent form to explicitly take part to the study and allowed the researchers to video-record them during the simulations. The teams underwent a two-day seminar about the implementation of the guidelines of the National Institute of Health about prevention and treatment of post-partum hemorrhage. Specifically, the topics treated during the seminar were:

- Guidelines about obstetric hemorrhage
- Clinical and organizational proactive approach to hemorrhage
- Clinical management of obstetric hemorrhage
- Clinical procedures for emergency management of obstetric hemorrhage
- The role of risk management for the proactive approach to risks
- The method of Significant Event Audit
- Non-technical skills and Crisis Resource Management
- Obstetric hemorrhage HFS (High Fidelity Simulations)

The seminar took place at the CISEF Gaslini, the International Centre for Studies and Training Germana Gaslini of Genoa. The simulator was the high fidelity NOELLE^®^ S574.100 Tetherless Maternal and Neonatal Birthing Simulator (Gaumard Scientific, Miami, Florida, U.S.A.). The scenarios were designed as the cases summarized in Table 2. All the scenarios were novel to the participants.

Each scenario lasted from 10 to 15 min and all the six teams took part of at least one of the simulations. All the participants (except the risk managers) were involved in at least one scenario. While the team was performing the simulation, the other teams observed the scenario using the CRM observational tool (Supplementary Material). The observers followed the scenario on wide screen in a separate room, in order to not disturb the simulation. The screen displayed the scene from two points of view (a distant camera capturing the whole team, and a close-up camera capturing the woman’s body, to see the details of maneuvers and actions performed on the simulator). The screen also and reported the clinical parameters of the woman and the fetus (heartbeat, oxygen peripheral saturation, non-invasive blood pressure). A team of simulation experts composed by nurses, anesthetists, midwives, gynecologists, and simulator technical support remotely controlled the simulator, both controlling the physiological parameters and the woman’s voice. In some scenarios a confederate played the role of the woman’s parent or partner attending the delivery. After the simulation, the debriefing was conducted by a practitioner with certified experience in simulation training and by a psychologist. They asked each participant to share what he/she had done in the scenario and reflect on the strengths and weaknesses of his/her behavior. The team risk manager was than involved in the debriefing in order to discuss procedural and organizational issues that emerged from the simulation. After that, the observers were asked to provide a peer-to-peer feedback using the CRM observational tool and explicitly referring to specific behavioral markers that were notable for the current scenario. The goal of the debriefing was to foster a proper metacognition about what they thought and why they took that specific course of action. Each observer, after the debriefing, rated the CRM observational tool on: (i) its usefulness in facilitating a reflection about one’s own professional practice and related thoughts (metacognition); (ii) its usefulness in helping the observation during the simulation and the peer-to-peer feedback, and (iii) it’s ease of use. All the ratings were on a 5-point rating scale (1 = “scarce”; 2 = “poor”; 3 = “average”; 4 =“moderate”; 5 = “extreme”).

## 3. Results

We collected a total of 101 observational forms. The first form from each participant was discarded, since presumably participants were still familiarizing themselves with the scale during the first rating; therefore, only 72 forms were analyzed for inter-rater reliability. Participants were asked after every form to rate the scale for usefulness and usability, but results only take in consideration the last rating given by each participant (N = 53). The frequency table the three usefulness and usability questions are presented in Table 3.

All the ratings had a median of 4, with 79% (metacognition), 79% (usefulness) and 66% (ease of use) of responses above the midpoint of the scale (interquartile ranges were 0, 0, and 1, respectively). A one-sample Wilcoxon test was used to determine whether or not the median was significantly higher than the midpoint of 3 for the items. All Wilcoxon tests were significant, with *p* < 0.001 for all items.

In order to investigate significant differences in distribution among the scores, we performed a paired samples Wilcoxon test. The only significant difference between scores is that between the rating of usefulness for a peer-to-peer feedback and the rating about the ease of use of the tool, V = 135; *p* = 0.017.

Item responses were analyzed for inter-rater reliability using a modified version of Fleiss’ Kappa for ordinal data not affected by Kappa paradoxes [27]. Inter-rater reliability was computed separately for each item, and results are reported in Table 4. Reliability was in the ‘fair agreement’ range (0.21–0.40) for all items except item 1, which had higher agreement (0.43).

## 4. Discussion

The ratings for usefulness and usability are skewed toward the upper part of the rating scale, which implies that the opinions of the participants were positive towards the tool. The CRM observational form was therefore considered a useful tool to trigger a reflection upon one’s own behavior and related thoughts (metacognition), a useful tool to provide specific feedback to the colleagues involved in the simulation, and a usable tool in general. Usability had the lowest rating, yet it was significantly higher than the average point (3). However, taking into account a high criterion for usability rating as suggested by [28], setting 4 as the acceptable rating for usability, we see that usability rating in our sample is significantly lower (mean value = 3.079) than four. The reason for this slightly lower rating could be due to the high number of data to be processed (reading all the items) in a short time (the colleagues returning from the simulation site to the debriefing room). The usability of the tool could be therefore improved by letting the observers familiarize themselves more with the items and providing them with more time to fill it in. In addition, the tool and the description of the CRM points had been provided as a booklet, for space reasons. We could find a better layout to fit the relevant information on a single page. However, we want to stress the fact that the participants had a short introduction to the CRM and the observation form prior to the simulation sessions. On average, they had been briefed in about 30 min. Despite this short time, the usability was nonetheless higher than the average point and we consider this a promising aspect of the tool, since it does not require a specific psychological expertise to be used and can become a suitable instrument for simulation-based training.

On the other hand, the high ratings of usefulness both for self-reflection and for peer-to-peer feedback are a promising sign that the tool can increase the learning potential of simulation. First of all, let us consider the CRM observational tool for peer-to-peer feedback. As argued by [29], the debriefing should focus on relevant actions observed in the scenario and help practitioners to elicit the background and often implicit cognitive and emotional processes that led to that action. By “relevant” we mean crucial for the explanation of the events, both effective and ineffective mental processes. A traditional attitude in training is to focus on what went wrong, pointing at the operators’ errors and teaching them the desired behavior or knowledge. However, this approach is limited for many reasons. First of all it is judgmental and could threaten the learning potential of simulation because of defensive reactions of the operators involved, which could justify their poor performance with the ecological limits and constraints of the simulator (e.g., “I don’t usually talk like that to a woman, this is a mannequin…”), the devices (e.g., “I did not know if the monitor was really working”), or the scene (e.g., “our delivery room is set up differently”). The CRM observational tool reports both effective and ineffective behaviors for each item of the CRM, therefore the observers are guided in their feedback towards the relevant actions of both sides of the performance continuum. Without the tool, the observers could be biased by the recollection of actions that fit with the judgmental attitude to search for the weaknesses of the practitioners. In addition, pointing at the mistakes is limited because safe performance is not just based on the reduction of mistakes, but in the increase and empowerment of the processes that led to good performance. The debriefing should not be focused on explaining what went wrong in the scenario, but on the process that let the team adapt to the critical situation, which skills were involved. Eliciting often latent and implicit dynamics, we can highlight the potential for safety and resilience of the team. Again, the CRM observational tool can help to this purpose, because the person conducting the debrief can decide to focus on the strengths of the team investigating the mental and social processes that led to the top rated items in the list.

Taking into account the high ratings of the tool as a good opportunity to reflect on one’s own behavior, we argue that the tool could increase the learning effect of observers and not only of the operators involved in the scenario, as demonstrated by [25]. The tool could enhance metacognition and a critically reflective attitude towards one’s own practice since it is based on specific behaviors that can be recollected from one’s memory to evaluate past activities, and can be kept in mind for the future. One typical characteristic of experts’ knowledge is that it is largely tacit, that is not easy to explicit verbally, nor to be fully aware of [30]. The debriefing aims at eliciting metacognition, critical reasoning, and self-reflective practice [31], and we argue that the observational tool based on observable and specific actions is a good trigger for these processes because it helps the user to focus on a specific behavior and to link it to an inner mental state.

Regarding inter-rater agreement, the results look promising, with fair agreement for all items of the scale. Nevertheless, the level of agreement is far from optimal; this study should be considered a step in the right direction, but refining the item content to make rating more objective should be considered a priority. Alternatively, short training sessions on how to use the scale could be a feasible way to raise inter-rater agreement. A short training session before using the scale could also raise the perceived ease of use of the scale itself.

## 5. Conclusions

This research aimed to develop an observational tool based on the CRM points proposed by Gaba and colleagues [10], adapted for the delivery room. One of the main goals of the present research was to fill in a gap in literature about CRM in simulation, where either CRM points are used as a guideline for the debriefing, but are often too general, or they are specified in terms of behavioral markers, but are not linked to the 15 points of CRM and are based on the non-technical skills frame of cognitive and social skills [6].

After an in-depth discussion with delivery room practitioners (anesthetists, gynecologists, midwives, and nurses) of the 15 items of the CRM list, we developed an observational tool inspired by the existing tools for the debriefing about non-technical skills. The observational tool for CRM in the delivery room was then composed by 19 items, and was administered to 53 practitioners (anesthetists, gynecologists, midwives, nurses, neonatologists, and risk managers). The tool was rated in terms of usefulness to trigger reflection on one’s own actions during everyday practice, usefulness to provide a peer-to-peer feedback after the simulation, and in terms of usability. All the three items received high ratings, indicating that the instrument was well received. The inter-rater agreement for each item was fair, suggesting that the scale can be considered a strong starting point, but should be refined in further studies.

Some of the limits of the present research concern the relatively lower rating of usability of the tool, probably due to the high cognitive load imposed to raters to fill the form in, which required a rapid thought about non-technical behaviors, a rather unusual task form many of them. Another limit of this study is that it was focused on self-reported ratings, but the validation of the tool will need further investigation in terms of inter-raters agreement, sensitivity, and coherence of the tool. A challenging aspect of the tool concerns the potential inapplicability of some CRM items to a specific scenario. For instance, the item about the use of cognitive aids states: “Checklists, manuals, tables, algorithms and/or expert consultations are used”. However, in some scenarios cognitive aids could be absent or not necessary and the item could be not applicable. In addition, some items refer to cognitive processes that may be difficult to observe. For instance, the item about the wise allocation of attention is rather abstract and was worded as “Team members explicit both a global picture of the case, and specific aspects of the situation”. This is an observable behavior pertaining an effective use of attentional resources, but it is not exhaustive of the wide range of “invisible” aspects behind this construct. Unfortunately this is the only aspect that can be listed in a behavioral marker tool.

A promising aspect of this tool concerns the involvement of the peers during the debriefing. As a consequence, the simulation becomes a learning activity not only for those involved in the scenario, but also for the colleagues watching the simulation. Training the simulation participants to use the tool could have the positive drawback of favoring a non-judgmental peer-to-peer feedback and, most of all, provide them with a take-home message based on a concrete, specific set of actions that will make their delivery room safer.

## Figures and Tables

**Table 1:** Sample of behavioral markers for Crisis Resource Management (CRM) in the delivery room (for the complete list see the Supplementary material).

Stem	Positive Anchor	Negative Anchor
*Know the environment*		
Resources (tools, personnel, materials)	are found and used when necessary	are found after looking around or after asking where they were
*Anticipate and plan*		
The potential clinical complications are discussed...	in advance	when they happen or are not discussed at all
*Call for help early*		
The request of medical and/or organizational resource supply is made...	as soon as the team members realize a problem has occurred	some after the problem has occurred
*Exercise leadership and followership with assertiveness*		
In the team…	someone is coordinating, assigning tasks, declaring the decisions	nobody is coordinating, assigning tasks, declaring the decisions
In the team…	the leader encourages and supports the opinions of the other colleagues	the others’ opinions are ignored, trivialized or discouraged
The team members…	share opinions and personal points of view	perform silently what required and do not express any personal opinion

**Table 2:** The three scenarios used in the simulation.

Main Clinical Issue	Participants	CRM Points Addressed
Post-partum hemorrhage due to cotyledon retention	2 Midwives Gynecologist Nurse anesthetist Anesthetist Relative (confederate)	Anticipate and plan Call for help early Use good teamwork Distribute the workload
Post-partum hemorrhage due to uterine atony	2 Midwives Gynecologist Nurse anesthetist Anesthetist Husband (confederate)	Anticipate and plan Use good teamwork Set priorities dynamically Re-evaluate repeatedly Crosscheck and double-check
Uterotonic drug management during peripartum hemorrhage	3 Midwives Gynecologist Anesthetist Midwife handing-over (confederate) Relative (confederate)	Anticipate and plan Call for help early Use good teamwork Distribute the workload Mobilize all available resources Use all available information Prevent and manage fixation error

**Table 3:** Descriptive statistics about usefulness and usability of the tool (N = 53).

Question	1	2	3	4	5 ^1^	Total
Usefulness for metacognition	1	0	10	29	12	52
Usefulness for peer-to-peer feedback	0	1	10	30	12	53
Ease of use	0	3	15	25	10	53

^1^ (1 = “scarce”; 2 = “poor”; 3 = “average”; 4 = “moderate”; 5 = “extreme”).

**Table 4:** Inter-rater agreement for each item (N = 72).

Item	Fleiss’ K ^1^	CRM Skill
ITEM 01	0.43 (0.33–0.54)	Know the environment
ITEM 02	0.27 (0.18–0.37)	Anticipate and plan
ITEM 03	0.23 (0.12–0.34)	Call for help early
ITEM 04a1	0.24 (0.09–0.39)	Exercise leadership with assertiveness
ITEM 04a2	0.28 (0.09–0.45)	Exercise leadership with assertiveness
ITEM 04b	0.28 (0.12–0.47)	Exercise followership with assertiveness
ITEM 05	0.35 (0.20–0.49)	Distribute the workload
ITEM 06	0.34 (0.20–0.49)	Mobilize all available resources
ITEM 07_1	0.21 (−0.01–0.40)	Communicate effectively—speak up
ITEM 07_2	0.30 (0.17–0.51)	Communicate effectively—speak up
ITEM 07_3	0.37 (0.31–0.42)	Communicate effectively—speak up
ITEM 08	0.28 (0.13–0.39)	Use all available information
ITEM 09	0.33 (0.24–0.44)	Prevent and manage fixation errors
ITEM 10	0.36 (0.22–0.47)	Crosscheck and double-check (never assume anything)
ITEM 11	0.25 (0.12–0.39)	Use cognitive aids
ITEM 12	0.30 (0.11–0.42)	Re-evaluate repeatedly
ITEM 13	0.33 (0.22–0.45)	Use good teamwork—coordinate with and support others
ITEM 14	0.25 (0.06–0.44)	Allocate attention wisely
ITEM 15	0.34 (0.17–0.44)	Set priorities dynamically

^1^ Fleiss’ kappa is a statistical measure to assess the agreement among raters on ordinal scale.

## Supplementary Materials

The following is available online at http://www.mdpi.com/1660-4601/15/3/439/s1, CRM Observational Tool.

## References

[1] Murray-Davis B., McDonald H., Cross-Sudworth F., Ahmed R., Simioni J., Dore S., Marrin M., DeSantis J., Leyland N., Gardosi J. (2015). Learning from Adverse Events in Obstetrics: Is a Standardized Computer Tool an Effective Strategy for Root Cause Analysis?. J. Obstet. Gynaecol. Can..

[2] Kaplan H.C., Ballard J. (2012). Changing practice to improve patient safety and quality of care in perinatal medicine. Am. J. Perinatol..

[3] Grobman W.A. (2012). Obstetric patient safety: An overview. Am. J. Perinatol..

[4] Kohn L.T., Corrigan J.M., Donaldson M.S. (2000). To Err is Human: Building a Safer Health System.

[5] Fung L., Boet S., Bould M.D., Qosa H., Perrier L., Tricco A., Tavares W., Reeves S. (2015). Impact of crisis resource management simulation-based training for interprofessional and interdisciplinary teams: A systematic review. J. Interprof. Care.

[6] Flin R.H., O’Connor P., Crichton M. (2015). Safety at the Sharp End: A Guide to Non-Technical Skills.

[7] Lucas A., Edwards M. (2017). Development of Crisis Resource Management Skills: A Literature Review. Clin. Simul. Nurs..

[8] White N. (2012). Understanding the role of non-technical skills in patient safety. Nurs. Stand..

[9] Messmer P.R. (2008). Enhancing nurse-physician collaboration using pediatric simulation. J. Contin. Educ. Nurs..

[10] Gaba D.M., Fish K.J., Howard S.K. (1994). Crisis Management in Anesthesiology.

[11] Gaba D.M. (2010). Crisis resource management and teamwork training in anaesthesia. Br. J. Anaesth..

[12] Fanning R.M., Goldhaber-Fiebert S.N., Undani A.D., Gaba D.M., Levine A.I., Schwartz A.D., DeMaria S., Sim A.J. (2013). Crisis Resource Management. The Comprehensive Textbook of Healthcare Simulation.

[13] Rall M., Gaba D.M., Miller R.D. (2005). Human performance and patient safety. Miller’s Anesthesia.

[14] Robertson B., Schumacher L., Gosman G., Kanfer R., Kelley M., DeVita M. (2009). Simulation-Based Crisis Team Training for Multidisciplinary Obstetric Providers. Simul. Healthc..

[15] Thomas M.J.W. (2017). Training and Assessing Non-Technical Skills. A Practical Guide.

[16] Kim J., Neilipovitz D., Cardinal P., Chiu M., Clinch J. (2006). A pilot study using high fidelity simulation to formally evaluate performance in the resuscitation of critically ill patients: The University of Ottawa Critical Care Medicine, High-Fidelity Simulation, and Crisis Resource Management I Study. Crit. Care Med..

[17] Ohland M.W., Layton R.A., Loughry M.L., Yuhasz A.G. (2005). Effects of behavioral anchors on peer evaluation reliability. J. Eng. Educ..

[18] Devcich D.A., Weller J., Mitchell S.J., McLaughlin S., Barker L., Rudolph J.W., Raemer D.B., Zammert M., Singer S.J., Torrie J. (2016). A behaviourally anchored rating scale for evaluating the use of the WHO surgical safety checklist: Development and initial evaluation of the WHOBARS. BMJ Qual. Saf..

[19] Watkins S.C., Roberts D.A., Boulet J.R., McEvoy M.D., Weinger M.B. (2017). Evaluation of a Simpler Tool to Assess Nontechnical Skills During Simulated Critical Events. Simul. Healthc..

[20] Mannella P., Palla G., Cuttano A., Boldrini A., Simoncini T. (2016). Effect of high-fidelity shoulder dystocia simulation on emergency obstetric skills and crew resource management skills among residents. Int. J. Gynaecol. Obstet..

[21] McConaughey E. (2008). Crew resource management in healthcare: The evolution of teamwork training and MedTeams. J. Perinat. Neonatal. Nurs..

[22] Miller LA. (2005). Patient safety and teamwork in perinatal care: Resources for clinicians. J. Perinat. Neonatal. Nurs..

[23] Haller G., Garnerin P., Morales M.A., Pfister R., Berner M., Irion O., Clergue F., Kern C. (2008). Effect of crew resource management training in a multidisciplinary obstetrical setting. Int. J. Qual. Health Care.

[24] Bracco F., Masini M., De Tonetti G., Brogioni F., Amidani A., Monichino S., Maltoni A., Dato A., Grattarola C., Cordone M. (2017). Adaptation of non-technical skills behavioural markers for delivery room simulation. BMC Pregnancy Childbirth.

[25] Lai A., Haligua A., Bould M.D., Everett T., Gale M., Pigford A.A., Boet S. (2016). Learning crisis resource management: Practicing versus an observational role in simulation training—A randomized controlled trial. Anaesth. Crit. Care Pain Med..

[26] Tan J.K.H., Sheps S.B. (1998). Health Decision Support Systems.

[27] Marasini D., Quatto P., Ripamonti E. (2016). Assessing the inter-rater agreement for ordinal data through weighted indexes. Stat. Methods Med. Res..

[28] Rosson M.B., Carroll J.M. (2001). Usability Engineering. Scenario-Based Development of Human-Computer Interaction.

[29] Dieckmann P., Patterson M., Lahlou S., Mesman J., Nyström P., Krage R. (2017). Variation and adaptation: Learning from success in patient safety-oriented simulation training. Adv. Simul..

[30] Engel P.J.H. (2008). Tacit knowledge and Visual Expertise in Medical Diagnostic Reasoning: Implications for medical education. Med. Teach..

[31] Josephsen J.M. (2017). A Qualitative Analysis of Metacognition in Simulation. J. Nurs. Educ..

