# Supplementary material for: Crisis Resource Management in the Delivery Room: Development of Behavioral Markers for Team Performance in Emergency Simulation
Source: Int J Environ Res Public Health. 2018 Mar 3;15(3):439. doi: 10.3390/ijerph15030439 (PMC5876984; doi:10.3390/ijerph15030439)
Supplement: Supplementary file 1 [file ijerph-15-00439-s001.pdf]

University of Genoa,
Department of Education Science

CRM Observational tool

1. Know the environment

One of the prerequisites of the CRM is to know the available resources and the characteristics of the specific work environment. The resources can be human or instrumental. It is important to know who to call for help, who is available in the several moments of the day, how to quickly contact these people and how long they will take to arrive. Concerning tools and instruments, it is necessary to know if they are available, where they are located, and how to operate them.

2. Anticipate and plan

Anticipation is the key of a goal-oriented behavior. In order to anticipate effectively, it is first necessary to take into account the case characteristics and plan the main steps. Anticipation allows practitioners to explore potential threats and critical situations. It's a matter of thinking ahead.

3. Call for help early

Realizing that you are having a difficulty and promptly asking for help is sign of good professional behavior (prudence, diligence and skill). Some clues to understand when it is necessary to call for help could be: when there are too many things to do, when you feel losing control over the situation, when problems get worse or are not reduced by the procedures or actions taken, or when you have no idea of what is going on.

4. A. Exercise leadership and followership with assertiveness

A team needs a leader, someone to take care of the task organization, tasks distribution, data gathering, and decision making about the main aspects of the situation. Leadership does not mean knowing more than the others, doing everything by oneself or submitting other people. It is rather a capacity to plan, decide and distribute tasks with effective communication.

B. Exercise followership with assertiveness

Followers are the key members of the team; they listen to the team leader and do what is necessary. That does not mean that they "turn their brain off": they should be able to sustain their point of view if they think their team leader is making a bad decision. They have the responsibility to make sure that the team leader takes into account their opinions and suggestions.

5. Distribute the workload

One of the main duties of the team leader is the distribution of workload. It is necessary that someone define the task to be accomplished and make sure that the tasks will be performed adequately. If possible, the team leader should be free from tasks to observe, gather information, and distribute activities. Every team member should be able to anticipate the leader requests, accomplishing tasks according to his/her competences. During emergencies, the team should to slow down and have a short brief about the situation in order to perform an effective planning, re-evaluation, and treatment.

6. Mobilize all available resources

Every resource, (human, technological, and organizational) that may help in coping with the problem should be taken into account and used. From the human point of view, the most relevant resources are the knowledge and skills of practitioners (together with the acknowledgement of one's own limit). Tools and instruments allow the translation of knowledge into action; some tools may be immediately available, while other need time to be mobilized.

© Fabrizio Bracco – www.unige.it
Michele Masini – VIE srl, www.vie-srl.com

7. Communicate effectively—speak up

Good teamwork is based on all the team members being on the same wavelength and being able to coordinate the effort. Communication is the link between members. Communication is effective only when the message has been received and understood.

8. Use all available information

It is necessary to integrate all the available sources of information. For instance, integrate immediately available sources (e.g., monitor, medical file, exams, colleagues, the patient), secondary sources (e.g., consultancy with other colleagues), and external sources (e.g., algorithms, protocols, the Internet).

9. Prevent and manage fixation errors

Human actions are based on a mental model of the current situation: if the model of the situation is wrong, it is possible that the actions will be wrong as well. The fixation error occurs when we use an inadequate mental model, notwithstanding the presence of clues in the situation that suggest alternative paths and solutions. The fixation error consists in the reiteration of an action or a clinical thought without taking into account alternative actions or ideas, while clues in the situation suggest a revision of the problem. This kind of mistake is very frequent in dynamic situations.

10. Crosscheck and double-check (never assume anything)

Cross-checking means to integrate different sources of information and revise what has been already done or what is going to be done. In general, it is better to give nothing for granted, performing multiple checks on relevant information.

11. Use cognitive aids

Resources like checklists, manuals, calculators, tables, algorithms, and consultancies from other colleagues have the same function: they enable the clinical-diagnostic-therapeutic reasoning. Cognitive aids support memory and enable practitioners to notice critical elements, especially under stress. Cognitive aids also help in following the best practices and guidelines, because during a crisis people tend to use previously learned and well-established procedures, even when they are no more updated and in line with the current guidelines/recommendations.

12. Re-evaluate repeatedly

Emergency medicine is dynamic. What is correct in a given moment could be wrong a minute later. Some parameters may gradually change over time, and small changes may be hard to notice. Checking the trend of parameters could help to notice a slow but insidious change. In addition, it is necessary to continually re-evaluate the situation, since the critical events have a dynamic nature.

13. Use good teamwork—coordinate with and support others

A short briefing before starting the task is time well spent to make the team more effective. Every team member should constantly support and care for others in order to coordinate team activities.

14. Allocate attention wisely

Human attention is a limited resource and multitasking is very difficult. For this reason, attention should be dynamically managed according to the priorities. For example, the memory technique "ABC = Airways, Breathing, Circulation", structured to distribute attention over time according to pre-defined sequences. Another strategy is to alternate the focus on the detail and on the global situation, delegating some activities to other qualified team members.

© Fabrizio Bracco – www.unige.it
Michele Masini – VIE srl, www.vie-srl.com

15. Set priorities dynamically

Dynamic situations require dynamic interventions. It is necessary to preliminarily set the priorities, while being able to constantly re-evaluate and modify them if necessary. What was wrong in a given moment may be the right thing to do in another moment. In addition, having a solution to a problem does not mean that it is the best solution or that there is only one way to solve it.

© Fabrizio Bracco – www.unige.it
Michele Masini – VIE srl, www.vie-srl.com

University of Genoa,
Department of Education Science

CRM Observational tool

Know the environment

Resources (tools, personnel, materials)…	are found and used when necessary	O O O O	are found after looking around or after asking where they were

Anticipate and plan

The potential clinical complications are discussed...	in advance	O O O O	when they happen or are not discussed at all

Call for help early

The request of medical and/or organizational resource supply is made...	as soon as the team members realize a problem has occurred	O O O O	some time after the problem has occurred

Exercise leadership and followership with assertiveness

In the team…	someone is coordinating, assigning tasks, declaring the decisions	O O O O	nobody is coordinating, assigning tasks, declaring the decisions
In the team…	the leader encourages and supports the opinions of the other colleagues	O O O O	the others' opinions are ignored, trivialized or discouraged
The team members…	share opinions and personal points of view	O O O O	perform silently what required and do not express any personal opinion

Distribute the workload

Task sharing is…	decided and communicated	O O O O	not clearly defined and/or communicated

Mobilize all available resources

All the human, technological and organizational resources are…	assessed and required	O O O O	not required

Communicate effectively—speak up

Communication is…	ordered and directed to specific members	O O O O	not addressed to specific members or chaotic (many people talking at the same time)

© Fabrizio Bracco – www.unige.it
Michele Masini – VIE srl, www.vie-srl.com

The person who gets an instruction…	confirms the reception by rephrasing it	O O O O	does not confirm its reception, or confirms without rephrasing it
Information is shared…	in a complete way (e.g., request of drug administration specifying active principle, quantity, way of administration, time for administration)	O O O O	in a incomplete of imprecise way (e.g., request of drug administration specifying just its name)

Use all available information

The situation is managed…	collecting information from the patient, the medical record, the protocols, and the team	O O O O	using just some of the available sources of information

Prevent and manage fixation errors

When the clinical action does not provide the expected outcome…	alternative data and solutions are searched	O O O O	no change is done

Crosscheck and double-check

The relevant data for the management of the situation (e.g., blood compatibility tests, drug administration procedures) are…	double-checked	O O O O	checked by a single operator or not checked at all

Use cognitive aids

Checklists, manuals, tables, algorithms and/or expert consultations are…	used	O O O O	not used

Re-evaluate repeatedly

Data useful for the management of the situation (clinical parameters, vital parameters on the monitors, etc.) are assessed…	explicitly and repeatedly	O O O O	only at the beginning of the scenario or re-evaluated without being explicitly communicated

Use good teamwork

Team members...	cooperate, independently by their role	O O O O	work independently from each other and without mutual help

© Fabrizio Bracco – www.unige.it
Michele Masini – VIE srl, www.vie-srl.com

CRM Observational tool

Allocate attention wisely

Team members…	explicit both a global picture of the case, and specific aspects of the situation	O O O O	are focused only on specific aspects

Set priorities dynamically

During the scenario…	priority re-evaluation is explicitly declared	O O O O	priorities are not re-evaluated

© Fabrizio Bracco – www.unige.it
Michele Masini – VIE srl, www.vie-srl.com
